# Pushing using real-time sonographic ultrasound education (PURSUE): A study protocol for a randomized controlled trial

**DOI:** 10.1371/journal.pone.0352315

**Published:** 2026-08-14

**Authors:** Gloria Anderson, Caterina Neri, Elvira Di Pasquo, Carmelinda Martino, Silvio Tartaglia, Michelangela Danza, Jessica Preziosi, Laura Beatriz De Fermo, Aurora Filippini, Miriam Anselmo, Valentina Sciabbarrasi, Chiara Sorrentino, Rosanna Miccoli, Martina Ottaviani, Roberta Di Battista, Tullio Ghi

**Affiliations:** 1 Technical Nursing Service Department, Fondazione Policlinico Universitario A. Gemelli, IRCCS, Rome, Italy; 2 Catholic University of the Sacred Heart, Milan, Italy; 3 Department of Obstetrics and Gynecology, Fondazione Policlinico Universitario A. Gemelli, IRCCS, Rome, Italy; University of Rwanda College of Medicine and Health Sciences, RWANDA

## Abstract

**Introduction:**

Maternal expulsive force is a key determinant of fetal head descent during the active second stage of labour. Pushing is most effective when abdominal muscle contraction is synchronised with relaxation of the levator ani muscle, thereby widening the pelvic hiatus. Trans-perineal ultrasound studies show that 15–20% of nulliparous women paradoxically contract the levator ani (co-activation) during pushing, narrowing the hiatus and slowing head descent. Consequently, about 20% of primigravidas experience a prolonged second stage, with higher rates of operative birth and perineal trauma.

**Methods and analysis:**

PURSUE is a single-centre, open-label, parallel-group RCT at Fondazione Policlinico A. Gemelli, Rome reported according to the SPIRIT 2013 and CONSORT 2010 guidelines. Nulliparous women between 28 + 0 and 37 + 0 weeks of gestation will be randomised 1:1 to midwife-led pushing training without ultrasound biofeedback or a midwife-led session combining theoretical instruction with real-time TPUS-guided pushing training. The primary endpoint is duration of the active second-stage of labour (minutes). Secondary outcomes include levator ani co-activation amplitude, spontaneous vaginal birth, perineal trauma, postpartum haemorrhage ≥ 500 mL, urinary/faecal incontinence at 6, 12 and 16 weeks postpartum and childbirth experience (CEQ-2). A sample of 228 (114 per arm) provides 80% power to detect a clinically meaningful reduction of 20 minutes(Cohen’s d = 0.37; α = 0.05, two sided). Allowing for 15% attrition, 268 women will be enrolled. The primary analyses uses multivariate linear regression adjusting for pre-specified covariates; secondary analyses are exploratory.

**Ethics and dissemination:**

The protocol is approved by Lazio Region Ethics Committee (Prot. 0000641/25) and registered on ClinicalTrials.gov (NCT07000240). Written informed consent is obtained at enrolment. An internal independent committee oversees safety. No formal interim analysis with pre-specified stopping rules is planned; accordingly, no alpha-spending adjustment is applied, and the pre-specified primary (confirmatory) analysis will be performed once, on the complete sample, after the end of follow-up. During the trial, preliminary results may be presented at scientific meetings for reporting and feasibility purposes; such presentations are exploratory and non-confirmatory, are not used to inform any decision to stop or modify the trial, and do not affect the pre-specified primary analysis or the control of the type I error rate. Results, the anonymised dataset and statistical code will be disseminated through peer-reviewed journals, conference presentations and an open-access repository within 12 months of completion.

**Trial registration:**

ClinicalTrials.gov NCT07000240

## Introduction

Fetal head descent during the active second stage of labour is influenced, among other factors, by maternal expulsive force. Optimal synergy occurs when abdominal‐muscle contraction is synchronised with relaxation of the levator ani muscle (LAM), which widens the pelvic hiatus and reduces soft-tissue resistance [1]. The pivotal role of the LAM in labour progress is well documented [2].

Trans-perineal ultrasound (TPUS) studies have shown that 15–20% of nulliparous women display paradoxical LAM co-activation, a contraction instead of the expected relaxation, during pushing [2, 3]. This phenomenon can be quantified on TPUS by tracking levator-hiatus diameter changes during the Valsalva manoeuvre [3]. Co-activation is associated with a higher fetal head station at term and a longer active pushing phase, increasing the likelihood of operative vaginal delivery, severe perineal tears, pelvic-floor damage and fetal distress [1,3,4]. These observations underscore the need for targeted educational interventions, such as real-time ultrasound biofeedback, to correct LAM co-activation and optimise pushing.

Pilot studies indicate that TPUS-guided, real-time visual biofeedback can shorten the second stage of labour by enhancing maternal neuromuscular coordination [5,6]; however, none has explicitly focused on promoting levator ani relaxation. Midwives are ideally placed to integrate TPUS into antenatal education and labour support, using visual demonstrations of pelvic-floor anatomy, fetal positioning and effective pushing techniques [7]. Combining TPUS feedback with midwife-led instruction therefore offers a personalised, evidence-based strategy to improve labour outcomes [8].

Therefore, the PURSUE (Pushing Using Real-time Sonographic Ultrasound Education) trial will evaluate whether a midwife-led, 60-minute session, combining an antenatal theoretical lesson on pushing with real-time TPUS-guided training, can shorten the second stage of labour and reduce the incidence of LAM co-activation in healthy nulliparous women compared with midwife-led pushing training without ultrasound biofeedback.

### Study hypothesis

Integrating real-time TPUS-guided training into routine midwife-led antenatal classes is expected to shorten the average duration of the second stage of labour by at least 20 minutes.

## Materials and methods

### Study design and duration

**Fig 1:**
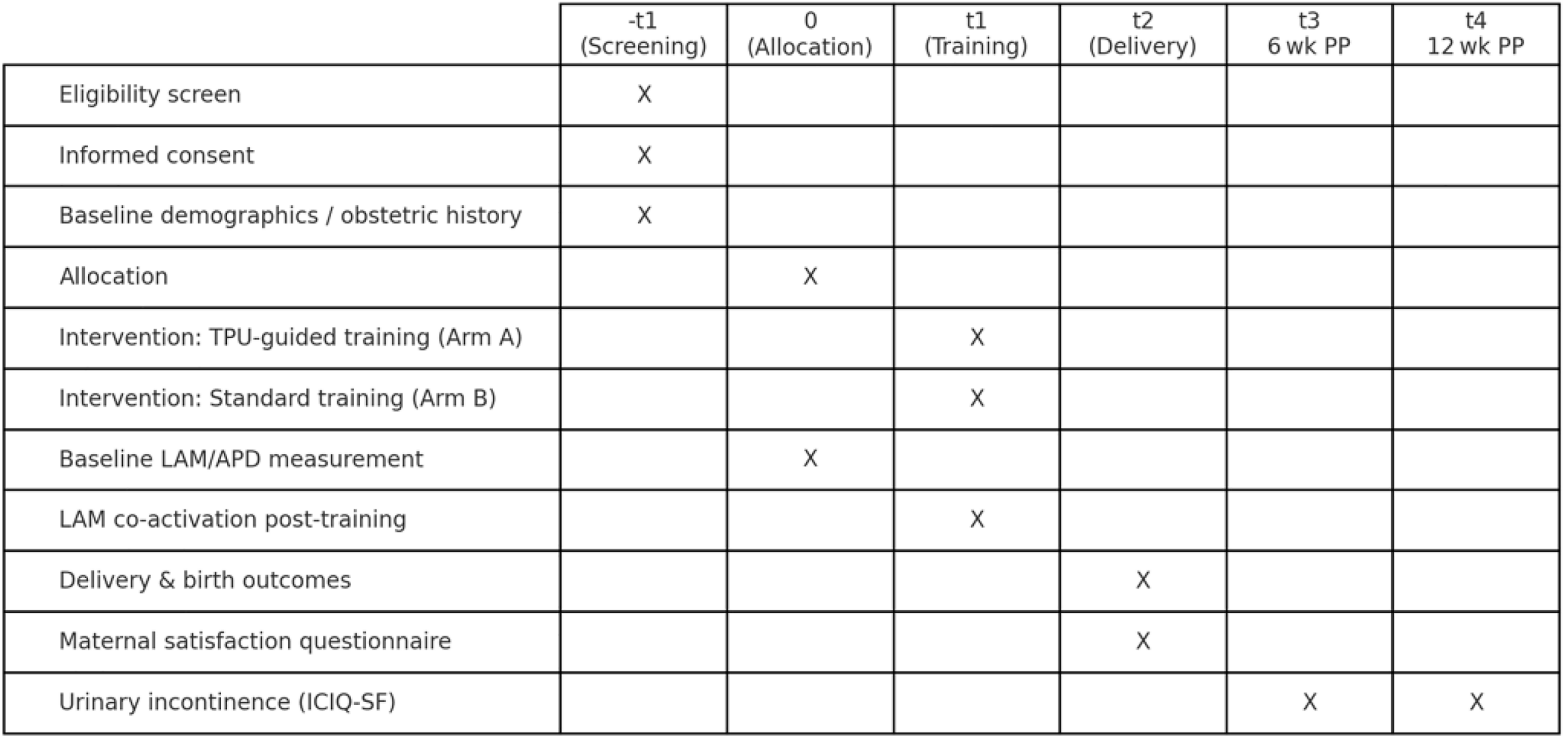
SPIRIT schedule of enrolment, intervention and assessment for the PURSUE trial.

This non-profit, single-centre, open-label randomised controlled trial (RCT) will be conducted at the Fondazione Policlinico Universitario Agostino Gemelli IRCCS, Rome, Italy. The protocol follows the SPIRIT 2013 statement; a completed SPIRIT checklist is provided as Supplementary File 2. Trial reporting will adhere to the CONSORT 2010 statement for parallel-group RCTs; a preliminary CONSORT checklist is provided as Supplementary File 1. SPIRIT schedule of enrolment, interventions and assessments provided in Fig 1. The enrolment period is from 18/07/2025–31/07/2026.

### Objectives

#### Primary.

To determine whether integrating real-time transperineal ultrasound-guided (TPUS) training into routine midwife-led antenatal classes shortens the average duration of the second stage of labour by ≥ 20 minutes compared with midwife-led pushing training without ultrasound biofeedback. In recognition of the multiple maternal, fetal and intrapartum factors that influence second-stage duration, overall efficacy will also be assessed using a composite maternal-neonatal outcome considering core endpoints recommended for obstetric trials [9] and previously used in large cohort studies and randomised controlled trials evaluating prolonged second-stage management [4,10].

*Secondaries:*

1. To quantify the proportion of women who demonstrate levator ani muscle (LAM) co-activation—defined as an anteroposterior diameter change (APD) < 0 mm—at two time-points: (i) during the TPUS-guided training session, and (ii) at birth, where an intrapartum sonographic assessment is feasible within routine clinical care.
2. To compare postpartum maternal satisfaction between the control and TPUS-guided intervention arms, as measured by the Childbirth Experience Questionnaire 2 (score range 0–100).
3. To quantify the difference of prevalence in 1st-, 2nd-, 3rd- and 4th-degree perineal tears between the control and the TPUS-guided intervention arms.
4. To measure the difference in total postpartum blood loss (mL) within 24 hours of delivery between the control and the TPUS-guided intervention arms.
5. To compare the rates of spontaneous vaginal birth, operative vaginal birth and unplanned caesarean sections between the control and the TPUS-guided intervention arms.
6. To evaluate the prevalence of stress or urge urinary incontinence at 6, 12, 16 weeks postpartum between the control and the TPUS-guided intervention arms, defined as an International Consultation on Incontinence Questionnaire-Urinary Incontinence Short Form (ICIQ-SF) score ≥ 6.
7. To compare the proportion of women that require pharmacological augmentation during the active second-stage of labour between the control and TPUS-guided intervention arms.
8. To quantify the association between persistent fetal malposition (non-occiput-anterior at full cervical dilatation and/or at delivery) and the length of the second stage of labour (minutes), and to test whether this association differs between the control and TPUS-guided intervention arms. This association is observational in nature and is not interpreted as a randomised comparison.

### Study population

Pregnant women who voluntarily participate in the prenatal education course offered by Fondazione Policlinico Agostino Gemelli IRCCS, Rome.

*Inclusion criteria:*

a) Nulliparous women.
b) Willingness to participate in the preparation training program on pushing techniques.
c) Singleton pregnancy.
d) Age 18 years or older.
e) Ability to understand and provide informed consent in Italian.
f) Plan to deliver at the Fondazione Policlinico Agostino Gemelli IRCCS.

*Exclusion criteria:*

a) Multifetal (e.g., twins, triplets) pregnancies.
b) Known obstetric complications or conditions that contraindicate a vaginal delivery (e.g., placenta previa).
c) Medical or psychiatric conditions that would prevent participation in the educational program or adherence to study protocols.
d) History of pelvic floor surgery or severe pelvic floor dysfunction that might affect participation or outcomes.
e) Inability to understand study requirements or provide informed consent.
f) Participants already enrolled in conflicting clinical trials or interventions that could influence the study’s outcomes.

#### Setting.

This single-center study will be conducted at Fondazione Policlinico Universitario Agostino Gemelli IRCCS Rome, an academic private tertiary-level hospital located in central Italy, with over 4,000 births per year. Childbirth care is characterised by continuous one-to-one intrapartum support provided by registered midwives under the supervision of senior midwives and obstetricians. Second-stage management follows physiological principles. In nulliparous women with epidural analgesia, delayed pushing is practised until the urge to push is felt or the fetal head reaches the pelvic floor; active pushing is then maternal-led, with verbal coaching by attending midwives. Practitioners do not routinely use ultrasound during the pushing stage to assess fetal station or perineal status. Intrapartum care is identical for both study arms; only the antenatal training component differs. In 2025 the hospital recorded an epidural analgesia rate of 72%, a primary cesarean section rate of 19%, and an operative vaginal delivery rate of approximately 11%, performed by vacuum extraction (Kiwi Cup).

Although a dedicated pelvic floor outpatient unit is available, no standardized postpartum pelvic floor rehabilitation protocol is in place. Rehabilitation sessions are therefore not scheduled as part of routine care, and attendance at external pelvic floor rehabilitation is not systematically recorded. All women are nonetheless advised at discharge of the importance of pelvic floor evaluation in the postpartum period. Attendance at pelvic floor rehabilitation will be recorded at the16 weeks follow-up contact. Should the rate of rehabilitation uptake exceed 10% in either arm, it will be included as a covariate in exploratory sensitivity analyses for pelvic-floor related outcomes.

### Study procedures

**Fig 2:**
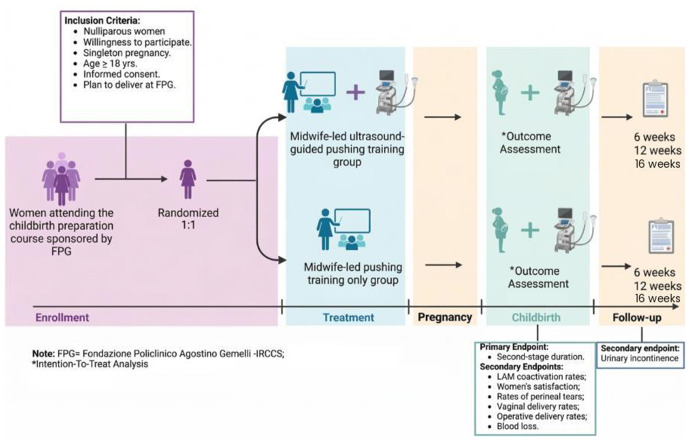
PURSUE study design.

Study procedures are summarized in Fig 2. Pregnant women between 28 + 0 and 37 + 0 weeks of gestation who participate in the antenatal preparation course sponsored by the Fondazione Policlinico Agostino Gemelli IRCCS will be informed about this study. The antenatal preparation course is held monthly, and it encompass nearly 60 women per course. Women who express interest will be scheduled for an in-person appointment with a research midwife, during which they will undergo eligibility screening. Those meeting the inclusion criteria and providing written informed consent will be randomized into one of two groups:

1. Midwife-led TPUS-guided pushing training group.
2. Midwife-led pushing training only group (control).

### Pushing Training Program

The pushing training program consists of a two-hour in-person session, structured as follows:

- 30 minutes of theoretical instruction, covering the physiological mechanisms of labour, the role of the LAM during pushing, and optimal pushing techniques.
- 30 minutes of practical exercises, during which women will be guided through breathing techniques, pushing strategies, and perineal relaxation.

**Fig 3:**
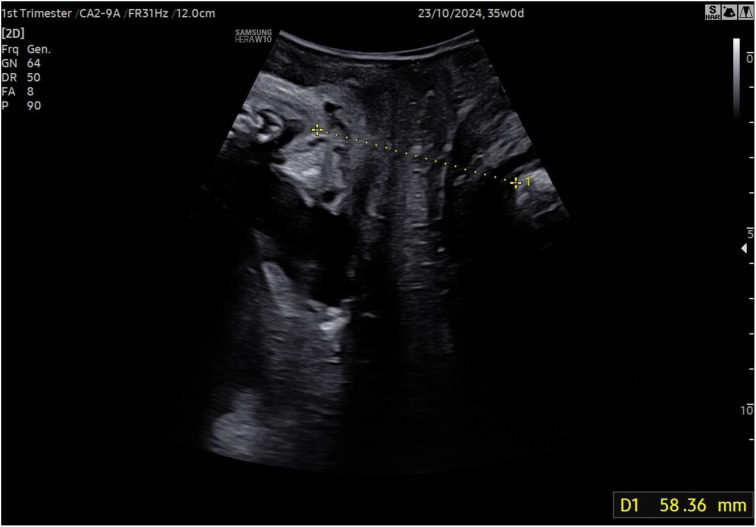
Transperineal ultrasound images illustrating measurement of anteroposterior diameter of levator hiatus, running from inferior border of pubic symphysis to anterior border of puborectalis muscle.

Women randomized to the TPUS-guided pushing training group receive additional TPUS evaluations during practical exercises, performed by a midwife trained in ultrasound imaging. The intervention is delivered by two midwives and three assistant midwife researchers. To ensure consistency and reduce inter-operator variability, the same midwife-assistant pair delivers the training to each allocated group throughout the study period. All midwives involved in the training received a minimum of one week of training on TPUS by a gynecologist with more than three years of experience in pelvic floor ultrasound. During training, all the midwives learned to perform TPUS in the midsagittal view with a Samsung HM70 EVO (Fig 3). Midwives learned to visualize pubic symphysis, fetal head, rectum and puborectalis muscle. To assess whether the LAM relax during the TPUS-guided pushing training, midwives measure the APD of the levator hiatus, from the posteroinferior border of the pubic symphysis to the anterior border of the puborectalis muscle (the main portion of the LAM), at rest and on maximum pushing manoeuvre, according to a previously described technique (Fig 3). During visual feedback, each woman watches the ultrasound screen to visualize the movement of the puborectalis muscle during pushing.

Midwives who delivered the educational intervention were different from those who provided intrapartum care during delivery, thereby minimising the risk of performance bias arising from knowledge of treatment allocation during labour. At the time of delivery, randomized women are instructed to push freely, following the techniques and guidance they previously received during the training sessions.

### Data collections

Data will be collected at multiple time points throughout the study to ensure comprehensive evaluation of the interventions’ effectiveness and safety (Fig 1). At enrolment, baseline demographic and obstetric information, including maternal age, parity, medical history, and pregnancy details, will be recorded. During the intervention period, attendance and engagement with both the online educational video and in-person sessions will be tracked. For participants in the TPUS-guided arm, detailed observations and measurements captured through TPUS will be documented during practical exercises to assess technique and muscle engagement.

Delivery outcomes will be extracted from medical records, including mode of birth (vaginal or caesarean section), duration of second stage of labour, incidence of any degree perineal tears, and any complications. Following childbirth, women’s satisfaction with the delivery experience will be evaluated using validated questionnaires. Women will be contacted at 6, 12 and 16 weeks post-partum. At each time point, urinary incontinence will be assessed using the ICIQ-SF questionnaire. At the 16 weeks follow-up, women will additionally be asked whether they have attended pelvic floor rehabilitation sessions since delivery. Collected data will be handled using a secure, de-identified database, with regular checks to ensure data integrity and adherence to ethical guidelines. Pseudonymized datasets will be stored on Zenodo (https://zenodo.org/), a secure open-access platform compliant with EU data protection regulations and FAIR principles.

### Randomization, blinding and treatment allocation

Once eligibility is confirmed and informed consent is obtained, participants will be randomised (1:1) into one of two study arms: using a computer-generated random sequence (“randomizeR” package in R**).** Assignment to study groups will be managed through sealed, opaque and sequentially numbered envelopes accessible only to the research midwife, thereby maintaining allocation concealment until the moment of assignment.

Given the nature of the interventions, this study is designed as an open-label trial; both the participants and the researchers will be aware of the group assignments. While full blinding is not feasible due to the involvement of TPUS in one arm, outcome assessors will be blinded to group allocation when evaluating outcomes and analysing data. This approach aims to reduce assessment bias and ensure that the treatment allocation does not influence the collection and interpretation of results.

### Sample size

The sample size was calculated to detect a mean reduction of 20 minutes in the duration of the active second stage of labour in the experimental group compared with the control group. Twenty minutes was pre-specified as the minimal clinically important difference for the duration of the active pushing phase, rather than as the anticipated magnitude of the treatment effect. Its clinical relevance is anchored to preliminary data showing that nulliparous women with LAM co-activation experience a longer second stage than those without co-activation (83 ± 63 vs. 63 ± 42 minutes, respectively). The pooled standard deviation is 53.5 minutes, yielding a standardised effect size (Cohen’s d) of 0.37, corresponding to a small-to-medium effect consistent with published trials of intrapartum ultrasound-based biofeedback interventions which were delivered intrapartum rather than antenatally. Using a two-sided independent-samples test with α = 0.05 and 80% power, the required sample size is 114 participants per arm (228 total). To account for an anticipated 15% attrition rate, encompassing study dropout, caesarean delivery before or during labour, and operative vaginal delivery precluding assessment of the primary endpoint, the final enrolment target has been set at 268 participants (134 per arm). We acknowledge that the hypothesised effect size assumes a biologically plausible benefit across the entire intervention group. Because preliminary data suggest that approximately 25% of nulliparous women exhibit LAM co-activation, the treatment effect may be attenuated in the overall population if benefit is concentrated in this subgroup. A pre-specified within-arm analysis by co-activation status at training is planned to explore this hypothesis (see Statistical Analysis Plan).

#### Statistical analysis plan.

Data will be analysed on an intention-to-treat basis. Baseline demographics, obstetric history, and pregnancy-related variables will be summarised by treatment arm using means and standard deviations (or medians and interquartile ranges) for continuous variables and frequencies and percentages for categorical variables. Between-group balance will be described using standardised mean differences (SMD); formal significance testing of baseline differences will not be performed, consistent with established guidance for randomised trials [11]. Two time intervals will be recorded for every woman who reaches the second stage of labour:(i) the duration of the active pushing phase, defined as the interval in minutes from the first coordinated maternal expulsive effort accompanying a uterine contraction, whether spontaneous or initiated following midwifery coaching, until delivery;(ii) the duration of the total second stage, defined as the interval in minutes from confirmed full cervical dilatation until delivery. The duration of the active pushing phase is the primary endpoint, referred to throughout as the duration of active second stage of labour**,** as it corresponds to the phase targeted by the intervention and to the phase in which levator ani co-activation has been shown to operate [2,3], and is the interval from which the assumed standard deviation used in the sample size calculation was derived. The duration of the total second stage is reported as a secondary endpoint. Both intervals are recorded contemporaneously in the electronic partogram and extracted for analusis by a researched blinded to allocation.

The primary endpoint will be analysed using multivariable linear regression, with treatment arm as the main predictor. The model will be adjusted for pre-specified baseline covariates only, that is, variables measured before randomisation: maternal age, body mass index at enrolment, gestational age at randomisation, maternal height, and sonographic estimated fetal weight at the last available third-trimester scan. Consistent with ICH E9, no post-randomisation variable is included in the confirmatory model, since conditioning on variables measured after allocation may invalidate the randomisation-based causal interpretation of the treatment effect. This applies in particular to fetal head position at full cervical dilatation, epidural analgesia and gestational age at delivery, which are potential intermediate variables on the causal pathway between the intervention and the primary endpoint. Their distribution across arms will be reported descriptively, and epidural analgesia and fetal head position will be examined as potential effect modifiers through interaction terms, as exploratory analyses. The primary result will be reported as an adjusted mean difference with 95% confidence interval; the p-value will be provided as secondary inferential information. If the residuals of the linear model indicate substantial departure from normality, a bootstrap confidence interval will be computed. Women who do not reach the active second stage (e.g., caesarean delivery before full dilatation) will not contribute to the primary analysis but will be retained in the ITT population for all secondary outcomes. For clarity, the primary estimand may therefore be summarised as the difference in adjusted mean duration of the active pushing phase between arms among randomised nulliparous women reaching the active second stage, with operative vaginal delivery and second-stage caesarean section handled under a treatment-policy strategy as delivery events at the observed time. The number of women contributing to each analysis, with the corresponding denominators and reasons for non-contribution, will be reported by arm in the CONSORT flow diagram, and the baseline characteristics of women not reaching the active second stage will be compared between arms using standardised mean differences. Sensitivity analyses will include multiple imputation under the missing-at-random assumption and inverse probability weighting to assess the robustness of the primary estimate to differential attrition, applied to observations that are missing rather than undefined, such as incomplete partogram times or non-returned questionnaires; where an imbalance is observed in the proportion of women reaching the active second stage, inverse probability of selection weights will be applied accordingly. Time to spontaneous vaginal birth from the onset of active pushing will additionally be described using cumulative incidence functions, with operative vaginal delivery and second-stage caesarean section treated as competing events rather than as censored observations, and compared between arms using Gray's test. This analysis is supportive and exploratory.

Mode of delivery (spontaneous vaginal birth vs. operative vaginal or caesarean), perineal tears (≥ 3rd degree vs. none/1st–2nd degree), postpartum haemorrhage (≥ 500 mL vs. < 500 mL), and need for pharmacological augmentation during the active second stage will be analysed using logistic regression with the same set of pre-specified baseline covariates. Results will be reported as adjusted odds ratios with 95% CIs, accompanied by absolute risks and risk differences by arm to facilitate clinical interpretation. For endpoints with a low expected number of events, Firth's penalised-likelihood estimation will be used. Third- and fourth-degree perineal tears will be analysed among all women reaching the second stage, with caesarean deliveries counted as no injury, and additionally among women delivering vaginally. Mode of delivery is also addressed within the competing-risks analysis described above. Total postpartum blood loss (mL) and maternal satisfaction (CEQ-2 score) will be analysed using multivariable linear regression as described for the primary endpoint, reporting adjusted mean differences with 95% CIs, with bootstrap confidence intervals reported for blood loss in view of its expected skewness. Urinary incontinence (ICIQ-SF score ≥ 6) will be assessed at 6 weeks, 12 weeks and16 weeks postpartum. The outcome will be modelled using mixed-effects logistic regression with a random intercept for participant and fixed effects for treatment arm, time point, and their interaction, adjusted for the same baseline covariates. This approach accounts for within-subject correlation across repeated assessments and allows estimation of the treatment effect trajectory over time and makes use of all available observations under a missing-at-random assumption. Both the interaction term and the adjusted treatment effect at each time point will be reported. Attendance at postpartum pelvic floor rehabilitation, a post-randomisation variable, will be summarised descriptively by arm and, where uptake exceeds 10% in either arm, included in sensitivity analyses for pelvic floor–related outcomes as described above. Pre-specified subgroup analyses will examine potential effect modification by: (i) Within-arm analysis of levator ani function (intervention arm only); (ii) gestational age at intervention (28–34 weeks vs. 35–37 weeks); and (iii) epidural analgesia (yes/no). Subgroup (ii) and subgorup (iii) are defined by post-randomisation variables and are reported as exploratory only. Subgroup effects will be estimated via interaction terms in the regression models and reported as stratum-specific effect estimates with 95% CIs. All secondary, subgroup, and repeated-measures analyses are regarded as exploratory and hypothesis-generating. The trial is powered exclusively on the primary endpoint. Results of secondary analyses will be reported as point estimates with 95% confidence intervals without correction for multiplicity and should be interpreted in the context of the overall pattern of findings rather than as confirmatory evidence. All analyses will be conducted using R (R Foundation for Statistical Computing) and STATA (StataCorp LLC).

### Ethical considerations

The study was approved by the Lazio Region Ethical Committee on 30 of April 2025 and received the internal approval by the scientific directorate of Fondazione Policlinico Agostino Gemelli IRCCS, Rome on 4 June 2025. The study will be conducted in full compliance with ethical guidelines, including the Declaration of Helsinki and local regulatory requirements. Prior to enrollment, all participants will receive detailed information about the study procedures, potential risks, and benefits, and will provide written informed consent. The privacy and confidentiality of women data will be strictly maintained, with data anonymized and securely stored. Participants will be informed of their right to withdraw from the study at any time without any impact on their standard care.

### Expected results

Introducing TPUS into a midwife-led pushing training program during prenatal courses is expected to significantly improve labour outcomes by reducing LAM coactivation and enhancing pushing effectiveness. Real-time biofeedback from TPUS can benefit both labouring women and their midwives. Midwives gain an objective, moment-to-moment guide for coaching effective pushing while helping to minimise levator ani co-activation; women, in turn, can see and feel when their pelvic floor is truly relaxing. Optimising the bearing-down effort in this way may shorten the second stage of labour, reduce resistance to fetal descent, and contribute to a smoother, more positive birth experience [3]. By reducing LAM coactivation, the PURSUE trial promotes more efficient pushing efforts, facilitating fetal head descent and shortening the active pushing phase.

Evidence supports that TPUS parameters, such as the angle of progression (AoP), are strong predictors of the duration of the second stage of labour. For example, an AoP greater than 160° is significantly associated with shorter remaining labour time in nulliparous women [12]. Moreover, visual biofeedback during pushing has been shown to improve coordination of pelvic floor muscle relaxation and contraction, which correlates with faster labour progression and higher rates of spontaneous vaginal delivery [5,13]. These improvements translate into a reduction in operative deliveries and cesarean sections, as TPUS use has been linked to lower rates of failed vacuum extraction and cesarean delivery without compromising neonatal outcomes [14].

Reducing the duration of the second stage of labour and minimizing pelvic floor muscle trauma are critical because prolonged labour is associated with increased maternal morbidity, including severe perineal tears and pelvic floor dysfunction [4]. TPUS-guided pushing training may help to avoid soft-tissue dystocia caused by LAM coactivation, thereby reducing the risk of pelvic floor injury. This protective effect on pelvic floor integrity could not only improve immediate birth outcomes but also supports better long-term pelvic health.

Finally, the incorporation of TPUS feedback into midwife-led prenatal education could enhances maternal satisfaction and confidence. Women receiving visual biofeedback report a greater sense of control and understanding of the pushing process, which positively influences their childbirth experience [15]. Empowering women with objective, visual information about their pelvic floor function and fetal progress could promote active participation in labour and may reduce anxiety related to pushing efforts.

## Supporting information

S1 File Protocollo_Pursue. (PDF)

S2 File SPIRIT-Checklist. (DOCX)
