# Supplementary material for: Pushing using real-time sonographic ultrasound education (PURSUE): A study protocol for a randomized controlled trial
Source: PLoS One. 2026 Aug 14;21(8):e0352315. doi: 10.1371/journal.pone.0352315 (PMC13475856; doi:10.1371/journal.pone.0352315)
Supplement: Supplementary file 1 [file pone.0352315.s001.pdf]

Pushing **U**sing **R**eal-time **S**onographic **U**ltrasound **E**ducation: A Randomized Controlled Trial

Version 1.0 of 14/02/2025

Summary

Financial disclosures *3*

Principal investigator(s) *3*

Abstract *4*

Introduction *5*

Material and methods *7*

Study design and endpoints 7

Study population 8

Study procedures 9

Data collections 10

Randomization, blinding and treatment allocation 10

Sample size 11

Statistical analysis plan 11

Ethical considerations 12

Expected results *12*

References *12*

Financial disclosures

The authors declare that they have not received any funding to support the current study.

Principal investigator(s)

Doctor Gloria Anderson, UOC SITRA, Fondazione Policlinico Agostino Gemelli IRCSS, Largo Francesco Vito 1

Prof. Tullio Ghi, Dipartimento di Ostetricia e Patologia Ostetrica, Fondazione Policlinico Agostino Gemelli IRCSS, Largo Francesco Vito 1

Doctor Roberta di Battista, UOC SITRA, Fondazione Policlinico Agostino Gemelli IRCSS, Largo Francesco Vito 1

Doctor Michelangela Danza, Università Cattolica del Sacro Cuore, Largo Fra Agostino Gemelli, 1, 20123, Milano

Doctor Rosanna Miccoli, Dipartimento di Ostetricia e Patologia Ostetrica, Fondazione Policlinico Agostino Gemelli IRCSS, Largo Francesco Vito 1

Doctor Caterina Neri, Dipartimento di Ostetricia e Patologia Ostetrica, Fondazione Policlinico Agostino Gemelli IRCSS, Largo Francesco Vito 1

Doctor Carmelinda Martino, Dipartimento di Ostetricia e Patologia Ostetrica, Fondazione Policlinico Agostino Gemelli IRCSS, Largo Francesco Vito 1

Doctor Elvira di Pasquo, Dipartimento di Ostetricia e Patologia Ostetrica, Fondazione Policlinico Agostino Gemelli IRCSS, Largo Francesco Vito 1

Abstract

This single-center, open-label, parallel-group randomized controlled trial aims to evaluate whether integrating transperineal ultrasound into a midwife-led pushing training program can reduce pelvic floor muscle coactivation during childbirth. Pregnant women in their second trimester, recruited from an online pre-childbirth course at Fondazione Policlinico Agostino Gemelli IRCCS, will be randomized to receive either standard midwife-led training or additional ultrasound-guided instruction. The primary outcome is the reduction in the duraction of the second stage of labour, with secondary outcomes including coactivation rates of pelvic floor muscles at delivery, maternal satisfaction, perineal tear rates, vaginal delivery rates, and postpartum urinary incontinence. With an estimated sample size of 136 participants accounting for a 15% dropout, the study anticipates that ultrasound-guided training will improve pushing efficiency, decrease pelvic trauma, and enhance delivery outcomes.

Introduction

Vaginal delivery is generally associated with fewer maternal complications (Zandvakili et al., 2017), quicker postpartum recovery (Adewale et al., 2023), and improved neonatal outcomes (Neu & Rushing, 2012) when compared to cesarean sections. Successful vaginal birth relies on effective pushing during the second stage of labor, which requires correct coordination of the muscles involved, particularly the pelvic floor muscles such as the levator ani (Lemos et al., 2017). However, many women may not have a clear understanding of optimal pushing techniques, which can lead to prolonged second stage of labor, increased maternal fatigue, and a higher likelihood of interventions including operative vaginal deliveries or cesarean sections (Lemos et al., 2017). Advances in medical imaging, especially ultrasound, have transformed obstetric care by providing real-time visualization of anatomical structures and physiological processes (Recker et al., 2024). The introduction of transperineal ultrasound offers a novel approach to teaching pregnant women the mechanics of effective pushing by visually demonstrating muscle movements and fetal positioning during simulated or actual pushing efforts (Kido et al., 2023).

The integration of ultrasound guidance into training programs has the potential to personalize and enhance the education process for expectant mothers. By directly observing how their pelvic floor muscles engage during pushing, women can adjust their techniques to maximize efficiency and safety (Lemos et al., 2017). This hands-on, visual feedback can lead to improved muscle coactivation, correct posture, and optimized force application, which are crucial for progressing labor and facilitating a vaginal delivery (Kamel et al., 2018; Youssef et al., 2019). Moreover, ultrasound guidance may help in identifying and correcting maladaptive behaviors or inefficiencies in pushing that are not apparent through verbal instruction alone. In litterature there is a lack of RCTs evaluating the effectiveness of such a targeted, ultrasound-guided training program on increasing vaginal delivery rates, reducing the duration of the second stage of labor, and minimizing the need for surgical interventions. The insights gained can inform clinical practice, leading to evidence-based recommendations for labor education and ultimately improving outcomes for both mothers and their newborns.

In Italy, midwives are legally recognized as autonomous healthcare professionals and are authorized to perform ultrasound examinations to support clinical decision-making, as

outlined by various national regulations (e.g., Legge n. 42/1999, Legge n. 251/2000, and the relevant professional guidelines). This legal framework acknowledges their competence in monitoring maternal and fetal well-being, enabling them to integrate ultrasound findings into the decision-making process during pregnancy and childbirth. Consequently, midwives can effectively utilize ultrasound imaging to assess the potential benefits of pushing training, ensuring evidence-based care and personalized support for expectant mothers.

Thus, we aim to evaluate the effectiveness of an ultrasound-guided training program on pushing techniques for pregnant women and its impact on the duraction of the second stage of labour and on vaginal delivery rates.

Objectives

Primary:

Its purpose is to evaluate whether incorporating transperineal ultrasound into a midwife-led training program on pushing techniques can reduce the duraction of the second stage of labour during childbirth for women attending prenatal classes.

Secondaries:

To measure the difference in rates of coactivation of the elevator muscle of the anus (LAM) in the experimental arm compared with the control arm

(b) To measure the difference in women's satisfaction with the childbirth experience and the training program in the experimental arm compared with the control arm

(c) To measure the difference in the rates of first-degree perineal tears in the experimental arm compared with the control arm

(d) Measure the difference in the the rate of second-degree perineal tears in the experimental arm compared with the control arm

(e) Measure the difference in the the rate of vaginal delivery in the experimental arm compared with the control arm

(f) Measure the difference nei the operative delivery rates in the experimental arm compared with the control arm

(g) Measure the difference in the rates of urinary incontinence at 6 and 12 weeks after delivery in the experimental arm compared with the control arm

Material and methods

Study design and duration

This study is designed as a single-center, open-label, parallel-group randomized controlled trial.

The study will last 24 months including 18 months for enrollment. The study will begin after the relevant ethics committee has issued a favorable opinion.

Endpoints

Primary Endpoint:

Reduction in the second stage of labor in the experimental arm compared with the control arm, defined as the elapsed time between complete cervical dilation and fetal expulsion.

Secondary Endpoints:

a) The levator ani muscle (LAM) coactivation rates were measured using transperineal ultrasound, evaluating the anteroposterior diameter (APD) of the levator hiatus at rest, during maximum pelvic floor muscle contraction, and during maximum Valsalva maneuver (both before and after visual feedback), with LAM coactivation defined as a reduction in APD on Valsalva compared to the resting state.

b) Women's satisfaction with the childbirth experience and with the training program, measured throught validated questionnaires

c) The rate of first-degree perineal tears, measured as the proportion of women experiencing superficial perineal lacerations involving only the vaginal mucosa and/or perineal skin without involvement of the underlying muscles, as documented in the delivery records.

d) The rate of second-degree perineal tears, measured as the proportion of women experiencing perineal lacerations involving the vaginal mucosa and perineal skin, extending into the perineal muscles but not involving the anal sphincter complex, as documented in the delivery records.

e) The rate of third-degree perineal tears, measured as the proportion of women experiencing perineal lacerations thatt extends into the muscle that controls the anus (the anal sphincter).

f) The rate of fourth-degree perineal tears, measured as the proportion of women experiencing perineal lacerations that extends further into the lining of the anus or rectum.

g) Vaginal delivery rates, measured as the proportion of women who achieved spontaneous or assisted vaginal birth (including vacuum or forceps-assisted delivery), excluding cesarean sections, as documented in the delivery records.

h) Operative delivery rates, measured as the proportion of women who required instrumental assistance for vaginal birth, including vacuum or forceps-assisted delivery, excluding spontaneous vaginal and cesarean deliveries, as documented in the delivery records.

i) Urinary incontinence rates at 6 and 12 weeks postpartum, measured as the proportion of women reporting involuntary leakage of urine during activities such as coughing, sneezing, or exertion (stress urinary incontinence) or experiencing a sudden, strong urge to urinate (urge incontinence), as assessed through clinical evaluation at follow-up visits.

l) Quantitative blood loss, defined as s the objective measurement of the volume of blood lost during and after childbirth.

Study population

Pregnant women in their second trimester who voluntarily participate in the pre-childbirth preparation course sponsored by the Fondazione Policlinico Agostino Gemelli IRCCS.

Inclusion criteria:

a) Nulliparous women in their second trimester (approximately 14–28 weeks of gestation).
b) Willingness to participate in the preparation training program on pushing techniques.
c) Singleton pregnancy.
d) Age 18 years or older.
e) Ability to understand and provide informed consent in Italian.
f) Plan to deliver at the Fondazione Policlinico Agostino Gemelli IRCCS.

Exclusion criteria:

a) Multifetal (e.g., twins, triplets) pregnancies.

b) Known obstetric complications or conditions that contraindicate a vaginal delivery (e.g., placenta previa).

c) Medical or psychiatric conditions that would prevent participation in the educational program or adherence to study protocols.

d) History of pelvic floor surgery or severe pelvic floor dysfunction that might affect participation or outcomes.

e) Inability to understand study requirements or provide informed consent.

f) Participants already enrolled in conflicting clinical trials or interventions that could influence the study's outcomes.

Study procedures

Pregnant women in their second trimester who participate in the pre-childbirth preparation course sponsored by the Fondazione Policlinico Agostino Gemelli IRCCS will be informed about this study. Women who express interest will be scheduled for an in-person appointment with a research midwife, during which they will undergo eligibility screening. Those meeting the inclusion criteria and providing written informed consent will be randomized into one of two groups:

1. Midwife-led ultrasound-guided pushing training group
2. Midwife-led pushing training only group

Pushing Training Program

The pushing training program consists of a two-hour in-person session, structured as follows:

- 30 minutes of theoretical instruction, covering the physiological mechanisms of labor, the role of the levator ani muscle (LAM) during pushing, and optimal pushing techniques.
- 30 minutes of practical exercises, during which women will be guided through breathing techniques, pushing strategies, and perineal relaxation.

Women randomized to the ultrasound-guided pushing training group will receive additional transperineal ultrasound (TPU) evaluations during practical exercises, performed by a midwife trained in ultrasound imaging. These assessments will ensure effective LAM relaxation during pushing practices and provide real-time visual feedback to optimize muscle coordination.

At the time of delivery, randomized women will be instructed to push freely, following the techniques and guidance they previously received during the training sessions.

Data collections

Data will be collected at multiple time points throughout the study to ensure comprehensive evaluation of the interventions' effectiveness and safety. At enrollment, baseline demographic and obstetric information, including maternal age, parity, medical history, and pregnancy details, will be recorded. During the intervention period, attendance and engagement with both the online educational video and in-person sessions will be tracked. For participants in the ultrasound-guided arm, detailed observations and measurements captured through transperineal ultrasound will be documented during practical exercises to assess technique and muscle engagement.

Delivery outcomes will be extracted from medical records, including mode of delivery (vaginal or cesarean), duration of second stage of labor, incidence of first-degree and second degree perineal tears, and any complications. Following childbirth, women's satisfaction with the training and delivery experience will be evaluated using validated questionnaires. Women will be contacted at 6 and 12 weeks post-partum to assess the urinary incontinence rates. Collected data will be handled using a secure, de-identified database, with regular checks to ensure data integrity and adherence to ethical guidelines. Pseudonymized datasets will be stored on Zenodo (https://zenodo.org/), a secure open-access platform compliant with EU data protection regulations and FAIR principles.

Randomization, blinding and treatment allocation

Once eligibility is confirmed and informed consent is obtained, participants will be randomized (1:1) into one of two study arms: the ultrasound-guided pushing training group or the standard midwife-led pushing training group. Randomization will be performed using a computer-generated random sequence to ensure allocation concealment and minimize

selection bias (“randomizeR”). Assignment to study groups will be managed through sealed, opaque envelopes or a secure electronic system accessible only to the research midwife, thereby maintaining allocation concealment until the moment of assignment.

Given the nature of the interventions, this study is designed as an open-label trial; both the participants and the researchers will be aware of the group assignments. While full blinding is not feasible due to the involvement of transperineal ultrasound in one arm, efforts will be made to blind outcome assessors to the extent possible when evaluating delivery outcomes and analyzing data. This approach aims to reduce assessment bias and ensure that the treatment allocation does not influence the collection and interpretation of results.

Sample size

The sample size was calculated based on preliminary data suggesting that women with levator ani muscle (LAM) coactivation post visual feedback experience a longer second stage of labor compared to those without LAM coactivation (83 ± 63 minutes vs. 63 ± 42 minutes, respectively). Assuming that the combination of obstetric ultrasound guidance and pushing training could reduce the second stage of labor by at least 20 minutes compared to pushing training alone, we estimated the required sample size using a two-sided test t with an alpha level of 0.05 and a power of 80%. The calculation yielded a sample size of 114 patients per group, 228 in total.

Statistical analysis plan

Baseline characteristics will be summarized by descriptive statistics.

The primary endpoint-that is, the reduction in the duration of the second stage of labor, defined as the time between complete cervical dilation and delivery-in the experimental group compared with the control group will be tested by applying Student's t test or otherwise the Mann-Whitney test.

Differences between groups in qualitative variables will be tested by applying the chi-square test or Fisher's exact test.

Secondary outcomes, including maternal satisfaction and incidence of first-degree perineal tears, will be analyzed by t-test, Mann-Whitney U test or chi-square test, depending on the type of variable and the distribution of data.

Two-way Anova will be applied to assess the difference in the two groups of urinary incontinence at 6 and 12 weeks after delivery.

All statistical tests will be considered statistically significant with $p < 0.05$, and analyses will be conducted using statistical software such as R, and STATA.

Ethical considerations

The study will be conducted in full compliance with ethical guidelines, including the Declaration of Helsinki and local regulatory requirements. Prior to enrollment, all participants will receive detailed information about the study procedures, potential risks, and benefits, and will provide written informed consent. The privacy and confidentiality of patient data will be strictly maintained, with data anonymized and securely stored. Participants will be informed of their right to withdraw from the study at any time without any impact on their standard care.

Expected results

It is anticipated that the incorporation of transperineal ultrasound into the midwife-led pushing training program will lead to a significant reduction in the coactivation rates of pelvic floor muscles during childbirth. This could translate into improved efficiency during the second stage of labor, a reduced risk of pelvic floor trauma, and potentially higher rates of uncomplicated vaginal deliveries. Secondary outcomes may include enhanced maternal satisfaction with the childbirth experience and a lower incidence of first-degree perineal tears. These findings could support the adoption of ultrasound-guided training protocols to optimize pushing techniques and improve maternal and neonatal outcomes.

References

Adewale, V., Varotsis, D., Iyer, N., Di Mascio, D., Dupont, A., Abramowitz, L., Steer, P. J., Gimovsky, M., & Berghella, V. (2023). Planned cesarean delivery vs planned vaginal delivery: a systematic review and meta-analysis of randomized controlled trials. *American journal of obstetrics & gynecology MFM*, *5*(12), 101186. https://doi.org/10.1016/j.ajogmf.2023.101186

Kamel, R., Montaguti, E., Nicolaides, K. H., Soliman, M., Dodaro, M. G., Negm, S., Pilu, G., Momtaz, M., & Youssef, A. (2019). Contraction of the levator ani muscle during Valsalva

maneuver (coactivation) is associated with a longer active second stage of labor in nulliparous women undergoing induction of labor. *American journal of obstetrics and gynecology*, *220*(2), 189.e1–189.e8. https://doi.org/10.1016/j.ajog.2018.10.013

Kido, K., Barbera, A., & Nagasaka, K. (2023). The role of transperineal ultrasonography for perinatal management: A review. *The journal of obstetrics and gynaecology research*, *49*(7), 1700–1709. https://doi.org/10.1111/jog.15645

Lemos, A., Amorim, M. M., Dornelas de Andrade, A., de Souza, A. I., Cabral Filho, J. E., & Correia, J. B. (2017). Pushing/bearing down methods for the second stage of labour. *The Cochrane database of systematic reviews*, *3*(3), CD009124. https://doi.org/10.1002/14651858.CD009124.pub3

Neu, J., & Rushing, J. (2011). Cesarean versus vaginal delivery: long-term infant outcomes and the hygiene hypothesis. *Clinics in perinatology*, *38*(2), 321–331. https://doi.org/10.1016/j.clp.2011.03.008

Recker, F., Gembruch, U., & Strizek, B. (2024). Clinical Ultrasound Applications in Obstetrics and Gynecology in the Year 2024. *Journal of clinical medicine*, *13*(5), 1244. https://doi.org/10.3390/jcm13051244

Youssef, A., Montaguti, E., Dodaro, M. G., Kamel, R., Rizzo, N., & Pilu, G. (2019). Levator ani muscle coactivation at term is associated with longer second stage of labor in nulliparous women. *Ultrasound in obstetrics & gynecology : the official journal of the International Society of Ultrasound in Obstetrics and Gynecology*, *53*(5), 686–692. https://doi.org/10.1002/uog.20159

Zandvakili, F., Rezaie, M., Shahoei, R., & Roshani, D. (2017). Maternal Outcomes Associated with Caesarean versus Vaginal Delivery. *Journal of clinical and diagnostic research : JCDR*, *11*(7), QC01–QC04. https://doi.org/10.7860/JCDR/2017/24891.10239
